# Microfluidic-Based Single-Cell Study: Current Status and Future Perspective

**DOI:** 10.3390/molecules23092347

**Published:** 2018-09-13

**Authors:** Haiwa Wu, Jing Zhu, Yao Huang, Daming Wu, Jingyao Sun

**Affiliations:** 1 Department of Pathology, College of Medicine, The Ohio State University, Columbus, OH 43210, USA; wu.680@osu.edu; 2 Department of Pharmaceutics, College of Pharmacy, The Ohio State University, Columbus, OH 43210, USA; syusejing@gmail.com; 3 College of Mechanical and Electrical Engineering, Beijing University of Chemical Technology, Beijing 100029, China; miamikissbye@outlook.com (Y.H.); eliasoaklane@yahoo.com (D.W.); 4 State Key Laboratory of Organic-Inorganic Composites, Beijing 100029, China; 5 Department of Chemical and Biomolecular Engineering, The Ohio State University, Columbus, OH 43210, USA

**Keywords:** microfluidics, single-cell study, polymeric, manufacturing

## Abstract

Investigation of cell behavior under different environments and manual operations can give information in specific cellular processes. Among all cell-based analysis, single-cell study occupies a peculiar position, while it can avoid the interaction effect within cell groups and provide more precise information. Microfluidic devices have played an increasingly important role in the field of single-cell study owing to their advantages: high efficiency, easy operation, and low cost. In this review, the applications of polymer-based microfluidics on cell manipulation, cell treatment, and cell analysis at single-cell level are detailed summarized. Moreover, three mainly types of manufacturing methods, i.e., replication, photodefining, and soft lithography methods for polymer-based microfluidics are also discussed.

## 1. Introduction

Cells are the basic units of the life activities of living organisms. Researches based on single-cell analysis are the foundation of bioscience. The differences between cells are always hidden by cell groups [1], while single-cell analysis can provide more precise and detailed information in specific cellular processes. Therefore, single-cell analysis is significant for the researches of cellular signal transmission, physiological pathology, and early diagnosis of serious diseases [2,3]. Since the early 1980s, capillary electrophoresis (CE) [4] has been successfully applied in single-cell analysis. Although CE can be used in the separation and detection of chemicals inside single cells, many procedures, such as cell manipulation and cell culture, are performed separately, which will raise costs and reduce the efficiency of the whole single-cell analysis process [5].

**Figure 1:**
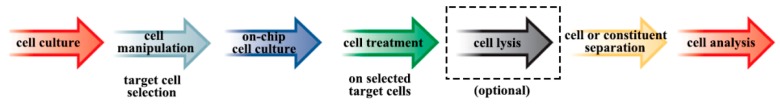
A collection of single-cell analysis on microfluidics from cell culture to cell analysis.

In 1990, Manz and Widmer first proposed the initial concept of the microfluidic [6]. Microfluidic systems, also called micro total analysis systems (μTAS) or “lab-on-a-chip”, have shown unique advantages in functional analytical processes [7]. Sequential operations like controlled transportation, drug delivery, mixing and dilution of chemical reagents, and cell manipulation and culture can be integrated in a single microfluidic device [8,9,10]. According to George Whitesides’ definition, microfluidics is “the science and technology of systems that process or manipulate small (10^−9^ to 10^−8^ L) amounts of fluids, using channels with dimensions of tens to hundreds of micrometres” [11]. Over the last two decades, researches based on microfluidics have attracted researchers from various fields and has become a hot topic. In addition, in the past few years, the microfluidic has attracted increased attention in the field of single-cell analysis, as microfluidic systems have the advantages of highly analysis efficiency, easy operation, small sample and reagent consumption, and dimension matching between channel sizes and cell diameter [12]. Figure 1 shows the collection of single-cell analysis on microfluidics from cell culture to cell analysis. Single-cell manipulation in the microfluidic system was realized for the first time in 1997 [13]. After that, cell culture [14], cell separation [15], component analysis [16], and many other operations of single-cell were realized one by one in the subsequent decade. Thus, the application of microfluidics has already become a significant driving force for discovery and advance in pharmacology, pathology, cell biology, and tissue engineering [17,18,19,20,21].

The application of different materials plays a crucial role in the construction of microfluidic systems. Materials with different properties, such as hardness, processability, electrical conductivity, transmittance, corrosion resistance, and biocompatibility, are the foundation of implementing different functions of microfluidic. Materials applied in the manufacturing of microfluidic can be divided into two main parts, one is inorganic materials like silica and glass, another part is polymer materials. Thus, polymer-based microfluidic is an important branch of microfluidic. Polymer materials have the advantages of variety, processability, and low cost [22]. By using appropriate material with specific properties, polymer-based microfluidic can offer great possibility and variety [23]. As distinguished from inorganic materials, such as silicon and glass, polymers are compatible with biological and chemical reagents [24,25]. Furthermore, polymers are much better for mass production with low cost, and therefore are the most commonly used materials for microfluidics. Therefore, this review will focus on the polymer-based microfluidic device for the single-cell analysis.

## 2. The Application of Polymer-Based Microfluidics for Single-Cell Analysis

Single-cell analysis is of great significance in early diagnosis, treatment, drug screening, and the research of cell physiology and pathological process of serious diseases. As microfluidics can meet the requirements of single cell monitoring, manipulation, treatment, and analysis under most conditions, it has already become a common method and a popular research area for single-cell analysis in recent years [26]. As complex procedures can be integrated into a single microfluidic system, microfluidics offer new opportunities for accurate, rapid, and low cost analysis with very small volumes [27]. Although the microfluidic system is a breakthrough with unique advantages for single-cell analysis, there are still two major obstacles to large-scale application. One is the manufacture and usage cost, another one is the applicability of microfluidics. In order to solve these obstacles, intensity efforts have been made by researchers around the world and the polymer-based microfluidic was selected as a promising solution [28]. On the one hand, polymer-based microfluidics can be cheap to manufacture in large quantities, which provide a way to transform this technology from its prototype in laboratory to large-scale commercial applications [29]. On the other hand, researchers can design specific microfluidics with selected polymer material to meet their unique requirements, as many polymers are compatible with chemical and biological reagents [30,31].

### 2.1. Cell Manipulation

**Figure 2:**
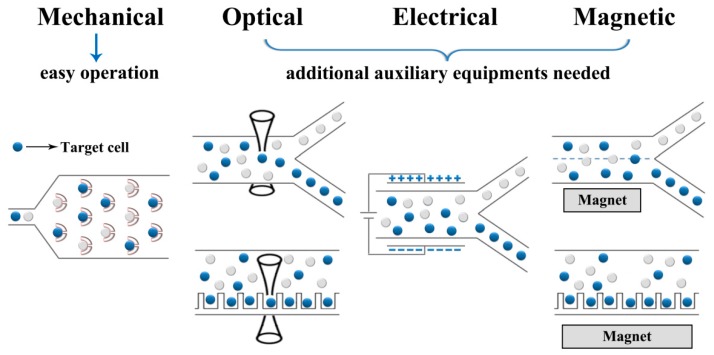
Illustrative schematics of mechanical, electrical, optical, and magnetic methods in cell manipulation techniques.

Cell manipulation is the first step in whole single-cell analysis process. In this step, single cells should be separated and then trapped or loaded in wanted positions for further analysis [32]. In terms of the different mechanisms, single-cell manipulation can be divided into several types: mechanical, electrical, optical, magnetic, and some other manipulation methods [33,34]. The illustrative schematics of these manipulation methods are shown in Figure 2. Recent years, the use of a polymer-based microfluidic combined with cell manipulation techniques is becoming more and more important in cellular information analysis at single-cell level.

Among these methods, the mechanical manipulation method is the most popular method due to its easy operation. With the help of specially-designed barrier structures and precise fluid control, people can realize different manipulations such as separation, sorting, trapping, and patterning on objective cells. Microgrippers [35], microwells [36], microfilters [37], microchambers [38], etc. are all common used barrier structures in mechanical cell manipulation. For example, VanDelinder and Groisman [39] described a cell and particle separation microfluidic device, and it was employed to separate white blood cells (WBCs) from whole human blood. The main body of this device was made of poly(dimethylsiloxane) (PDMS) sealed with cover glass, which was very simple and affordable to fabricate. Lee’s group also have made many efforts on developing mechanical cell manipulation techniques. They designed a U-shaped trapping microstructure array on a PDMS microfluidic with surface modification and the whole single cell trapping process could be done within 30 s [40]. Some combinations of mechanical manipulation microfluidics with fluid dynamic effects and physical measuring, e.g., deformability, were also developed in recent years [41,42]. Di Carlo’s group introduced a PDMS-based microfluidic system that can achieve the goal of high-throughput label-free cell sorting and enrichment. Utilizing the balance between deformability-induced and inertial lift forces of passing cells in the microchannel, cancer cells were sorted and enriched based on cell size and deformability [43]. Guo et al. presented a SU-8 microfluidic system fabricated with the photolithography technique. With the help of micropipette aspiration, single cell deformability was precisely measured in this polymer-based microfluidic system [44].

Comparing between size-based and mechanically selected cell manipulation, electrical, optical, and magnetic manipulations always need some additional auxiliary equipment or devices to obtain much more targeted and precision operation on objective cells. Nevertheless, the benefits from the abilities such as displacement measuring on the nanometer level, force applying on picoNewton level, and contactless manipulation, these techniques have also become promising tools for single-cell researches [45].

Among these techniques, optical manipulation using optical tweezers can provide high resolution in single-cell trapping. A PDMS microfluidic system integrated with optical tweezers and image processing equipment was proposed by Wang et al. for cell sorting. Sorting experiments of yeast cells and human embryonic stem cells (hESCs) were performed to prove its effectiveness [46]. For the purpose of time-resolved single-cell behavior monitoring under changing chemical and physical environments, a combination of microfluidics, fluorescence microscopy, and optical tweezers was developed and tested using yeast cells. In this system, optical tweezers were applied for cell selection and positioning [47].

Besides optical manipulation, other contactless manipulation methods including electrical and magnetic manipulation for single-cell sorting were also developing rapidly in recent years. The dielectrophoresis (DEP) technique utilized the motion of dielectric particles in nonuniform electric field to realize the separating, patterning, and other operations on target cells [48]. Hu et al. reported a microfluidic device made from photosensitive polyimide (PI) using the photolithography technique. An electrokinetic sorting methodology was developed to perform the DEP process in microchannels, and rare target cells were enriched in a single sorting round [49].

Hydrogels were also applied to fabricate polymer-based microfluidics. Matsue’s group fabricated a Gelatin methacrylate (GelMA) microfluidic device, and 3D microscale organization was successfully established within it using a cell patterning technique with DEP forces [50]. Furthermore, Liu et al. developed a permalloy and polystyrene-based microfluidic device with micropillar structures to generate the magnetic flux density peak. Jurkat cells were automatically captured and aligned onto the microstructure array area [51]. Polymer-based microfluidics with well-defined microstructure for direct and continuous separation of circulating tumor cells (CTCs) from blood cells was also widely researched. A permanent magnet was integrated with microchannels to generate gradient magnetic field in these microfluidics [52,53].

### 2.2. Cell Treatment

Cell treatment is an important procedure before cell analysis and its integration with microfluidic devices will help the development of research techniques [54,55,56]. As cell treatment is a broad concept that contains many kinds of operation, we only focus on several most common types in this review. Microfluidic cell culture is necessary for in vitro experiments. Cell lysis is indispensable for cell constituent analysis. Cell electroporation is also a typical on-chip operation and hot topic in microfluidics. So in the following section, a brief introduction and discussion of cell culture, cell electroporation, and cell lysis will be presented with selected examples.

Comparing with traditional macroscopic culture methods, such as cell culture in dishes, well-plates, and flasks, microfluidic cell culture shows significant advantages [7,57,58,59]. For example, a well-designed microfluidic cell culture can satisfy the needs of individual cell types and cellular co-cultures at the same time [60]. Besides the flexible design of microfluidic devices for cell culture, microfluidic cell culture is much better for the mimic of cells’ natural microenvironments. Moreover, microfluidic cell culture can improve the efficiency of high throughput experiments with greater operation control and less contamination risk. Less reagents cost due to a lower application amount in the forming of the microenvironment is also an advantage of microfluidic cell culture. Nowadays, the rapid development of microfluidic cell culture opens the door for real time on-chip analysis with low numbers of cells or even single cells [61,62]. Vedel et al. developed a PDMS microfluidic device to study the role of intercellular interactions and single-cell properties. By changing cell density, neighboring cells will significantly influence the migratory behavior (direction, speed, and displacement) of an observed single cell [63]. To culture primary hippocampal neuronal/glia cells in an isolated but synaptically connected environment, Robertson et al. fabricated a microfluidic device containing adjacent chambers with connected microchannels and an individual inlet and outlet. It provided an in vitro test platform for the research of synaptic activity modulation and connectivity within mixed primary hippocampal co-cultures [64]. Wang’s group presented an integration of polymer-based microfluidic cell culture and a cell-based biosensor for the detection of physiological parameters in the cell. This application made the sensitive and rapid analysis of biomedicine possible with small amounts of reagents and small numbers of cells down to the single-cell level [65,66,67,68]. Furthermore, because PDMS has the advantages of low toxicity, easy shaping, and high thermal stability and biocompatibility, it has already become one of the most widely used polymer material in microfluidics. Whiteside’s group had already studied the influence of PDMS on the growth of several types of cells and no significant effect was observed [69].

**Figure 3:**
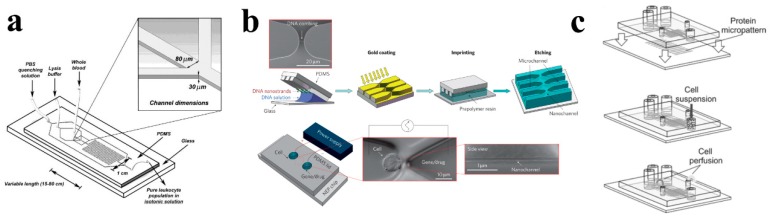
(**a**) Schematic of on-chip cell lysis process [75]. Reproduced with permission from Palaniappan Sethu; et al. Analytical Chemistry; published by American Chemistry Society, 2004. (**b**) Schematic of poly(dimethylsiloxane) (PDMS)-based microfluidic for nanochannel electroporation [84]. Reproduced with permission from Pouyan E. Boukany; et at. Nature Nanotechnology; published by Springer Nature, 2011. (**c**) Micropatterned microfluidic system for long-term cell cultures [86].

Various microfluidic devices have been proposed for cell lysis in recent years, such as chemical lysis [70], mechanical lysis [71], electrical lysis [72], laser lysis [73], thermal lysis [74], and so on. Among them, chemical and mechanical based cell lysis microfluidic devices are the most commonly used and studied. The schematic of the on-chip cell lysis process is shown in Figure 3a [75]. A microfluidic device with a microwell array for single-cell chemical lysis was fabricated using PDMS by Jen et al. recently. Cells and lysis buffer were pumped into the microchannel and the lysis process of single cells was examined with a fluorescence microscope. This method was suitable for high-throughput cell lysis as the cells can be fully lysed within 12 s [76]. Madou’s group developed a microfluidic system for mechanical cell lysis on micro- and nanoscales. Their novel microfluidic system was an integration of a circular plastic disk, magnetic field, cell centrifugation, and lysate clarification device. Lysis efficiency up to 65% was achieved using this technique [77].

The combination of microelectrodes and microchannels or microstructure arrays within microfluidic devices is the foundation of on-chip cell electroporation [78,79]. Among different applications of microfluidic electroporation, in vitro cell transfection is the most widely used one [80,81,82]. On-chip electroporation within limited space can obtain the necessary electric field with much lower voltage than traditional methods, which significantly avoid the potential risks of high voltage and excess heat. This technique can be applied to the transfection for both primary cells and cell lines [79,83]. Recently, nanochannel electroporation (NEP) (as shown in Figure 3b) was first proposed as an improvement of on-chip cell electroporation technique by Lee’s group in Ohio State University [84,85]. Combing with optical tweezers, a PDMS microfluidic device was fabricated for NEP process at the single-cell level. By adjusting the pulse number and duration time, single cells located at one nanochannel between two microchannels can be delivered with precise amounts of transfection agents (dose control) through the nanochannel without affecting cell viability.

Tourovskaia and coworkers fabricated a microfluidic perfusion system suitable for long-term (>2 weeks) culture of muscle cells that spanned the entire differentiation process from myoblasts to myotubes [86]. Figure 3c showed the schematics of the device assembly, cell seeding, and cell perfusion of their microfluidic system for cell culture. Cell-adhesive surface microdomains alternating with a robust cell-repellent coating mimic in vivo spatial cues for muscle cell assembly, while the microfluidic system provided precise control of biochemical components surrounding the cells and their perfusion rates. Observing results showed no differences in differentiation between microfluidic and traditional cultures. Thus, this method can be used as an improved in vitro model to study muscle cell differentiation and related characterizations.

### 2.3. Cell Analysis

As there are a great variety of cell analysis technologies, we will mainly focus on the three most important and familiar parts and their combination with microfluidic systems in this section. They are flow cytometry (FC), biochemical sensing, and whole cell assay techniques, respectively.

**Figure 4:**
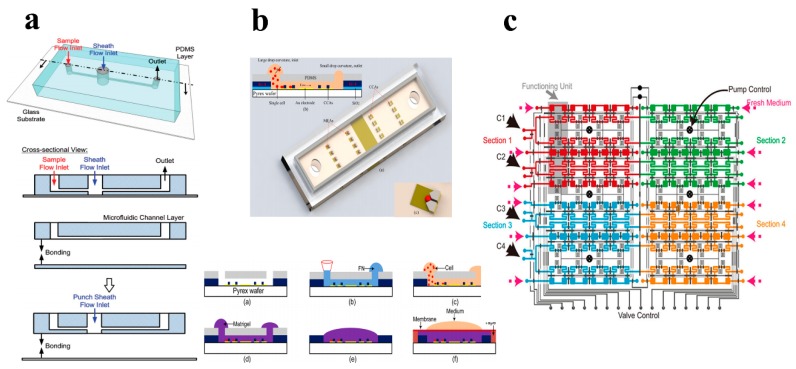
(**a**) Schematic of a PDMS-based flow cytometry microfluidic device and its fabrication process [94]. Reproduced with permission from Royal Society of Chemistry; published by Royal Society of Chemistry, 2012. (**b**) Schematic of an on-chip biochemical sensor [107]. Reproduced with permission from Tien Anh Nguyen; et al. Analytical Chemistry; published by American Chemical Society, 2013. (**c**) The layout of a very complicated microfluidic device for whole cell assay [108].

FC is a useful method to measure the chemical and physical characteristics of different kinds of cells [87,88,89,90]. According to the advantages of quantitative detection, various protein feasibility and high throughput, FC has become the most successful and widely used technique for single-cell analysis [91,92,93]. Figure 4a shows the schematic of a PDMS-based FC microfluidic device and its fabrication process [94]. Over the years, the development of polymer-based microfluidics drives from the FC devices evolving from ‘benchtop’ to integrated chips, making them much portable and affordable [95,96].

For example, Tung’s group presented a PDMS-based microfluidic device for the drug testing and FC analysis of uniform sized tumor spheroids. The formed and cultured tumor spheroids were introduced into the microfluidic device using human hepatocellular carcinoma cells (HepG2). Three anticancer drugs were applied to tumor spheroids, and microfluidic FC were utilized to provide post-treated information in single cell resolution, which made it easier to compare the drug functions on tumor cells in more in vivo-like 3D situation [97]. Wu et al. also developed a microfluidic FC device for protein analysis at single-cell level. The analysis of TLR4 receptor activation, phosphorylation of ERK1/2, and the detection of TNFα in LPS stimulated macrophages were successfully performed using their microfluidic device [98].

With the rapid development of microfluidic devices and its advantages of reagents manipulation controllably, it is showing more and more attractive potential in the detection of cell metabolites and the analysis of intracellular parameters [99,100,101]. It is called microfluidic biochemical sensing and can be applied at even a single-cell level [102,103]. Peng et al. reported a PDMS-based microfluidic device for the detection of intracellular calcium mobilization and fluorescein diacetate (FDA) at single-cell level. In their work, the variation of PH and glucose and the retaining of single cells within microchannels were all controlled by 3D liquid flow [104,105]. They further developed a CD-like microfluidic device with spiral microchannels for DNA hybridizations under similar principle and manipulation [106]. Nguyen et al. presented a polymer-based microfluidic device integrated with so-called “cell-substrate impedance sensing (ECIS)”. This sensor chip (as shown in Figure 4b) provided a rapid and selective detection method for single cancer cell migration in 3D extracellular matrixes, which can be applied for cancer research [107].

Whole cell assay is also an important part of cell analysis. The layout of a very complicated microfluidic device used for the whole cell assay is shown in Figure 4c [108]. While low biochemical quantity in a single cell would influence the analytical accuracy of biological variation, the advances of microfluidic techniques significantly increased the potential of whole cell assay with high throughput [109,110,111]. Bang et al. performed a PDMS-based microfluidic device, which can realize drug diluting precisely with a buffer solution with serially increasing concentrations, for cytotoxicity tests in a reproducible and efficient manner [112]. Huang’s group developed a microfluidic device for high throughput transcriptome sequencing. The microfluidic device was applied for minimizes contamination and excellent detecting sensitivity and measuring precision to prepare cDNA from single cells [113].

The small size (~10 μm), tiny cell component content, and numerous types of cells greatly increased the difficulty of single-cell manipulation and analysis. Therefore, in order to carry out single-cell studies, analytical methods must be able to handle very small volumes, determine a variety of compounds simultaneously, and can give good qualitative and quantitative results. In order to meet these requirements, some improvements of the microfluidic devices for single-cell study are necessary. First, the size of channels on microfluidics should be within the certain range of 10 to 100 μm to achieve dimensional compatibility with signal cell. Second, build more three-dimensional channels in microfluidic devices for more inner space and easier manipulation. Third, microfluidic devices are more suitable for flat geometry design, which is much suitable for observation during detection and analysis. Last but not least, proper surface treatment is of great importance in obtaining the optimum testing performance and prolonging the service life of microfluidic devices for single-cell study.

### 2.4. Applications of Single-Cell Analysis on Microfluidic Platforms

Single-cell analysis can provide accurate information about intracellular substances and biochemical reactions. Moreover, it can reflect the specific relationship between cell functions and chemical components, as well as the special role of certain cells in living organisms. Different kinds of single-cell analysis (e.g., single cell RT-PCR, single cell multiplex protein detection, single cell transcriptomics, etc.) have already been done by researchers around the world using microfluidics as a new type of testing platform.

**Figure 5:**
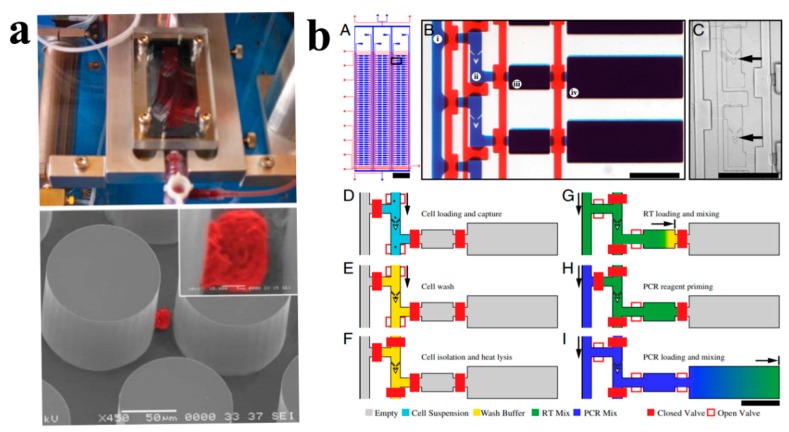
(**a**) Specially designed microfluidic device can capture cancer cell (pseudo colored red) from whole blood in single step [114]. Reproduced with permission from Sunitha Nagrath; et al. Nature; published by Springer Nature, 2007. (**b**) Design and operation of the microfluidic system for single-cell high-precision RT-qPCR gene expression analysis [115].

Nagrath et al. presented a unique microfluidic platform capable of efficient and selective separation of viable circulating tumor cells (CTCs) from peripheral whole blood samples in a single step (as shown in Figure 5a) [114]. CTCs are cancer cells isolated via liquid biopsy of the blood and can be phenotypically and genetically characterized to provide key information for guiding cancer treatment. Flow velocity and shear force were found to be two essential parameters that determine the efficiency of cell capture. The high sensitivity (1 target cell in 1 billion blood cells), selectivity (higher than 47% purity), and yield (99%) of their microfluidic device made it an ideal platform for real-time monitoring of response to cancer therapy.

The development of an integrated technology for scalable analysis of single-cell transcription is a long-sought milestone in microfluidic techniques. Hansen’s group in Princeton University proposed a fully integrated microfluidic device (as shown in Figure 5b) that can perform high-precision RT-qPCR gene expression analysis on hundreds of single cells per run [115]. The microfluidic system featured six sample input channels, each divided into 50 compound reaction chambers for a total of 300 RT-qPCR reactions. After over 3300 single-cell experiments, they are convinced that microfluidic RT-qPCR is suitable for quantitative analysis of SNV detection and miRNA expression, which are difficult by alternative hybridization methods. Wu’s group [116] proposed a high-content microfluidic real-time platform by combining single-cell quantitative real-time PCR (qRT-PCR) together with high-throughput arrays as a powerful tool for comparative study of the regulation of development processes in single cells. This high-throughput single-cell qRT-PCR provides a standardized comparative analysis of the mechanism of self-renewal and differentiation of human pluripotent stem cells.

Shahi and coworkers proposed a new microfluidic-based technique for single-cell multiplex protein detection [117]. Its feasibility was demonstrated by characterizing surface proteins of different cell types at the single-cell level and distinguishing between the cells by their protein expression profiles. DNA-labeled antibodies offer a variety of advantages for analyzing proteins in a single cell, including the ability to amplify low-abundance markers so that they can be sequenced, quantified using molecular indices, and almost limitless multiplexing. Even single-cell transcriptomics could be performed via microfluidic systems. Kirschner’s group at Harvard Medical School developed a high-throughput droplet-microfluidic approach for barcoding the RNA from thousands of individual cells for subsequent analysis by next-generation sequencing [118]. This method showed surprisingly low noise distribution and is easy to adapt to other sequencing-based analyses. Mouse embryonic stem cells were analyzed by researchers, revealed in detail the population structure and the heterogeneous differentiation initiation after leukemia inhibitory factor (LIF) withdrawal. These high-throughput single-cell results allowed researchers to deconstruct cell populations and infer gene expression relationships.

## 3. Common Manufacturing Methods for Polymer-Based Microfluidics

The primary fabrication methods of microfluidics were well-explored and established by the semiconductor industry. Hence, silicon and glass used to be the dominant materials for the fabrication of microfluidics, when the microfluidic technique was still in the stage of initial development in the early 1990s [119]. However, although silicon-based materials were very attractive, the disadvantages, such as high producing cost, narrow application range, fragile and so on, drove producers and researchers to seek more suitable materials. 

With the rapid development of microfluidics in recent years, polymers seemed to be the best optional material for the manufacturing of microfluidics. For researchers, the wide range of polymer materials, which allowed them to choose the most appropriate material for their specific applications, was extremely attractive. For example, some kinds of polymers, e.g., PDMS, are compatible with biological and chemical reagents, which offered the possibility to apply microfluidics to the fields of pharmacology, pathology, cell biology, and so on. For commercial producers, compared to silicon and glass, polymer-based, especially plastic-based microfluidics, showed many benefits including simplified manufacturing procedures and low cost. These two superiorities of polymer material laid a solid foundation for the large-scale application of microfluidic devices in our daily life. Although polymer-based microfluidics have several advantages, as previously mentioned, and can meet the requirements of single-cell operations listed in the previous section, they still has several limitations based on the physical and chemical properties of polymer materials. For example, most polymers have poor corrosion resistance and are easily attacked by the solvent applied for single-cell analysis, which can lead to stress cracking phenomena and destroy the whole microfluidic system. Polymer materials (e.g., polymethyl methacrylate and PMMA) have poor mechanical properties and low surface hardness, meaning that scratches might occur during the operation processes of single-cell studies, reducing the service life of microfluidics. Moreover, the development of surface modification methods for polymer materials still lags, with many existing problems. This limits the application of microfluidic for single-cell study especially when researchers have special surface treatment requirements.

**Figure 6:**
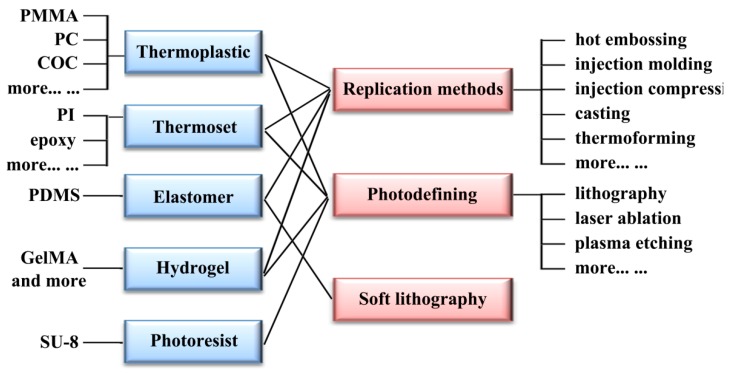
Illustrative schematic of the relationship between different kinds of polymer materials and manufacturing methods for polymer-based microfluidics.

This section will introduce currently relevant manufacturing methods for polymer-based microfluidics. As mentioned above, there are several groups of polymers that can be applied to microfluidics, including thermoplastics, thermosets, elastomers, and hydrogels. Figure 6 presents an illustrative schematic of the relationship between different kinds of polymer materials and manufacturing methods for polymer-based microfluidics. Addressing different polymer materials, replication methods like injection molding and hot embossing, photodefining methods like lithography and laser ablation, and soft lithography for elastomers like PDMS will be discussed, respectively.

### 3.1. Replication Methods

Replication methods take an important position in the manufacturing process of polymer-based microfluidics. In a manner of speaking, the advantages of high-throughput production, being both suitable and low cost, are two principal causes for the enormous commercial achievement of polymer materials in price-sensitive markets. From the beginning of the twentieth century, the development of replication methods has led to a huge progress in the field of micro- and nanofabrication [120,121,122]. Thus, replication methods have also become the foundation for the commercialization of polymer-based microfluidics. The basic characteristic of these methods is the application of a mold with micro/nano structures on its surface. The desired structures are transferred from the molds onto a polymer under the appropriate temperature, pressure, and other parameters. Among different replication methods for the manufacturing of polymer-based microfluidics, injection molding and hot embossing are two most commonly used types [123,124]. Although soft lithography for elastomers is also a kind of replication method in some sense, it will be discussed individually later due to the dominant role of PDMS in the field of microfluidics for cell analysis [125].

The injection molding process is the most widely used manufacturing method for polymer materials on the macroscale. Hence it is not surprising that, in the trend of micro/nano fabrication since the beginning of the twentieth century, it also became one of the most widely used manufacturing methods. The first publication that presented polymer-based microfluidics prepared with the injection molding method was published in the 1990s [126]. Although this technology has some limitations due to its high precision requirements of the mold and overall equipment [127], it still plays a crucial role in the commercialization process of polymer-based microfluidics.

**Figure 7:**
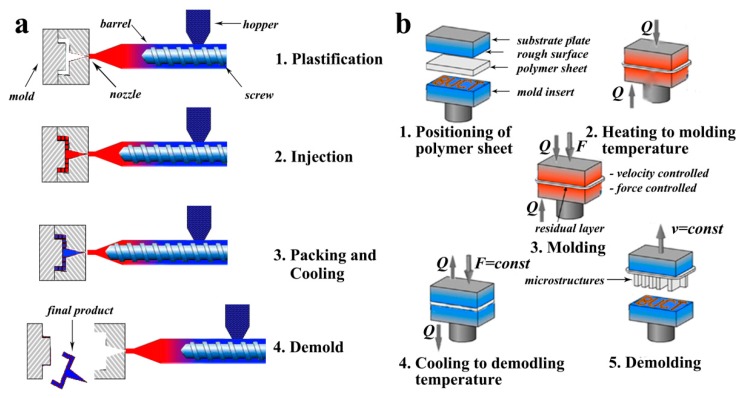
(**a**) Schematic diagram of an injection molding machine and the major steps of the injection molding procedure. (**b**) Schematic diagram of a hot embossing machine and the major steps of the hot embossing procedure.

A schematic diagram of an injection molding machine and the major steps of injection molding procedure is shown in Figure 7a [128]. It can be seen that an injection molding machine is composed primarily of an injection unit and clamping unit. Briefly speaking, a single complete cycle of injection molding process can be divided into five steps [129]. In the first step, predried polymer granules are fed into the barrel through hopper. Secondly, polymer granules are melted in the heating barrel, and transferred to the nozzle with the help of a metering screw. In this step, the temperature inside the heated barrel is an important parameter, since it should be higher than the melting temperature and lower than the thermal decomposition temperature of the specific polymer material used. Then, the melting polymer is injected into the microstructured mold under relatively high pressure. In order to get better replication results, vacuum-assisted injection molding, injection compression molding, and many other kinds of injection molding methods were developed [130,131]. Fourthly, after the melting polymer is fully filled into the microstructured mold, it should be cooled below the solidification temperature of the polymer within mold for structure definition. Finally, the microstructured products like microfluidic devices are demolded from the mold and the next cycle can be started. Researchers also have made many efforts to find better processing conditions for micro-injection molding. For example, Fu et al. used the Taguchi method to optimize the processing conditions of the micro-injection molding process. The width and depth of microchannel were tested in cross-sectional profiles and seemed as indicators for the evaluation of replication rate. The closest geometrical parameters of microchannel to the microstructure on mold were obtained after optimization [132]. Furthermore, many researchers mentioned that barrel temperature, injection velocity, holding pressure, and mold temperature were the most common and important processing conditions for injection molding [133].

In addition to injection molding, hot embossing is also a widely used method for the fabrication of polymer-based microfluidics both in industry and academia [134,135]. The advantages of comparatively simple process and high availability for a large range of materials lead to its rapid developing in the last two decades. Up to now, three different molding principles of hot embossing have been successively developed, including Plate to Plate (P2P) [134,136,137], Roll to Plate (R2P) [138], and Roll to Roll (R2R) [139,140], to meet the increasing demand for high volume production of microstructured devices [141,142].

Figure 7b shows a schematic diagram of a hot embossing machine and the major steps of hot embossing procedure. In most cases, a typical micro hot embossing process is composed of five major steps [143]: (a) positioning of polymer sheet, (b) heating of a semi-finished product, a thin polymer foil, to molding temperature, followed by (c) an isothermal molding by embossing (velocity- and force-controlled), (d) the cooling of the molded part to demolding temperature, with the force being maintained, and finally (e) demolding of the component by opening the tool. Cheng et al. applied the hot embossing process to amorphous polymers. Polycarbonate (PC), poly(methyl methacrylate) (PMMA), and polystyrene [38] microfluidics with cavity dimensions around 50 μm, 100 μm, and 200 μm, respectively, and a thickness of 2 mm were fabricated. The influences of several processing conditions, such as cavity dimension, compression temperature, and embossed materials, on the replication rate were investigated in detail [144]. Mathur et al. successfully transferred the microstructures from a silicon-based mold to PMMA substrates. They produced various microchannels with and without pillars, and the optimum processing parameters were demonstrated to be 100 °C embossing temperature and 10 kN applied force [145]. Besides, hot embossing methods with high replication accuracy to the range of tens of nanometers have also been named nanoimprinting, which is a potential technique for future lithography processes on nanoscale [146,147].

### 3.2. Photodefining Methods

In recent years, photodefining methods have also became one set of frequently used technologies for the fabrication of polymer-based microfluidics [148,149]. Photodefining methods utilize light such as ultraviolet (UV) light and laser to motivate the reactions, like polymerization and evaporation of a polymer material. Among different kinds of photodefining methods, photolithography and laser ablation are two typical manufacturing methods for polymer-based microfluidics [150,151].

**Figure 8:**
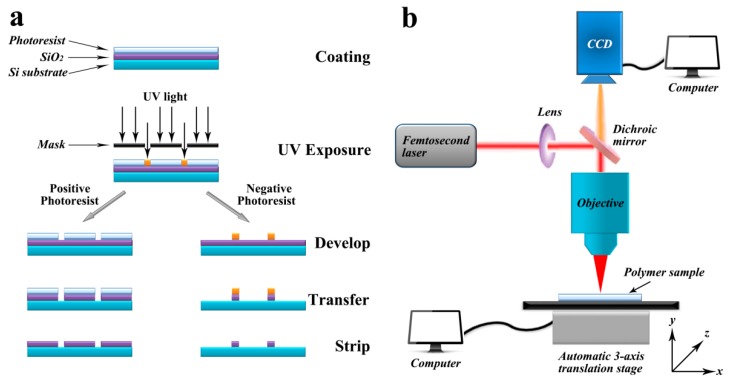
(**a**) Schematic diagram of photolithography process using positive or negative photoresist. (**b**) Schematic diagram of a three-dimensional laser ablation machine.

Photolithography, also called UV lithography or optical lithography, is a method to pattern micro- and nanostructures on different substrates. The schematic diagram of the photolithography process using positive or negative photoresist is shown in Figure 8a. This method uses light to motivate the polymerization reaction and transfer geometric patterns through photomasks, which can build solid microstructures out of a liquid resist. The most widely used photoresist is SU-8, which was developed for the simplification of the microfabrication process in the 1990s. Benefiting from the strong crosslinking during the UV exposure process, SU-8 shows significant advantages, such as high thermal, chemical, and mechanical stability. It also leads to the widespread application of SU-8 in the field of microfluidics both in industry and academic [152]. The technical procedures of a typical SU-8 photolithography process can be described as follows. Firstly, the light sensing material, i.e., SU-8, is spin-coated or simply dropped onto the substrate, most commonly a silicon wafer. Secondly, target patterns are structured under UV exposure with the help of a photomask. After the uncured photoresist is washed-out, open microchannels, which can be used in cell culture microfluidics or other cell experiments, are obtained. If microfluidics with close microchannels are required for further cell analysis, then the third step will be needed to close and the open SU-8 microchannels. Laminate films such as PDMS, polypropylene (PP), and polyethylene (PE), and even another film of dry SU-8 can be used to carry out the sealing process [153]. Meanwhile, a second resist layer built by another photolithography process is also an alternative to create close microchannels [154].

Metz et al. developed a sacrificial layer method for the fabrication of microfluidics. They used SU-8 and PI to build close microchannels and focused on the decomposition of the sacrificial materials inside them. This method provided a high efficiency and versatile technique for the manufacturing of polymer-based microfluidics [155]. As a combination of replication and photolithography methods, the so-called “UV-nanoimprinting lithography” (UV-NIL) process was first proposed by Grant Willson and his group in 1999 [156]. In recent years, it has become a commonly used method for the manufacturing of polymer-based microfluidics. Chen et al. presented a flexible microfluidic device for the detection of Salmonella in liquid samples. The UV-NIL process was performed with a R2R manufacturing system to build high accuracy microchannels on PET films. A sealing process was also used to form a closed microfluidic device [157].

Laser ablation is also a rapid manufacturing method for the fabrication of polymer-based microfluidics and the schematic diagram of a three-dimensional laser ablation equipment machine is shown in Figure 8b [158,159]. In laser ablation process, high energy laser beam is focused onto the surface of polymer and the material at the focal point will be evaporated. Open microchannels or other designed microstructures can be established with the movement of laser beam or polymer substrates in x and y direction. Besides, these geometries also can be built by exposing polymer substrates through a mask. A 193 nm ArF excimer laser [160], 248 nm KrF excimer laser [161], and 308 nm XeCl excimer laser [162] are three typically used pulsed lasers, while lasers with different wavelengths will be suitable for different amounts of materials. After ablation, the debris generated during laser ablation process should be completely removed from the microstructures. Suriano’s group utilized the femtosecond laser ablation technique to fabricate polymer-based microfluidics. The chemical and physical properties of cyclic olefin polymer [120], PMMA, and PS microchannels were systematically researched. They found that only PMMA microfluidics can remain transparent, while COP and PS were significantly dehydrogenized and oxidized during the laser ablation process. This means that PMMA was the only viable thermoplastic polymer for the fabrication of microfluidics by femtosecond laser ablation among these three polymer materials [163].

### 3.3. Soft Lithography

Generally speaking, the soft lithography process [164] is a combination of molding, printing, and embossing techniques. It is especially suitable for the fabrication of micro- and nanostructures on nonplanar and even 3D substrates at low cost. Basing on these three different operations, a large number of soft lithography techniques such as replication, microcontact printing (μCP) [165], microtransfer molding (μTM) [166], solvent-assisted micromolding (SAMIM) [167], micromolding in capillary (MIMIC) [168], and many other emerging methods [169] were successively developed. Nevertheless, soft lithography replication is the only method used for the manufacturing of polymer-based microfluidics among the methods mentioned above. As PDMS microfluidic devices play a crucial role in the field of cell analysis, we discuss each device, in detail, individually.

**Figure 9:**
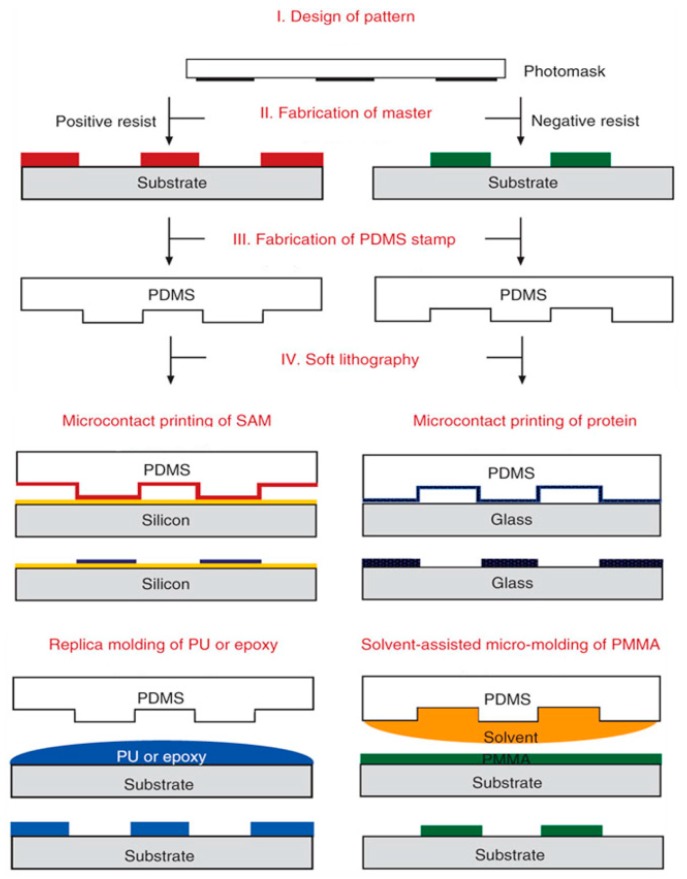
Schematic illustrations of the major steps involved in soft lithography and several major types of soft lithography techniques [170].

The schematic illustrations of several major types of soft lithography techniques are shown in Figure 9 [170]. The soft lithography process for the fabrication of PDMS microfluidic device can be described as follow. Firstly, replication, photodefining, or other methods are performed to fabricate a microstructured mold. Secondly, a casting of PDMS prepolymer is made on the surface of the microstructured mold and is then cured on hot plate under vacuum condition to fully replicate. Thirdly, holes are punched in the cured PDMS to use as inlets and outlets for introducing and collecting fluids. Then, the PDMS is treated with a laminate film (e.g., PDMS, silicon wafer, glass, PMMA, PP, etc.) and an oxygen plasma cleaner for oxidation, which is helpful for sealing process. Finally, a conformal contact is formed between the treated surfaces of the PDMS and laminate film to build the final product of microfluidic device. With the help of the soft lithography replication method, Hung et al. developed a high aspect ratio microfluidic device with a microchamber array for cell culture. Human carcinoma (HeLa) cells were cultured with continuous perfusion of medium at 37 °C using this device, which provide a high throughput platform for quantitative research in the field of cell biology [171].

## 4. Conclusions and Future Directions

Single-cell analysis is one of the focus areas of analytical chemistry, which is closely related to human health. Meanwhile, microfluidic is a typical interdisciplinary area that is closely related to chemistry, physics, life science, computer science, material science, and so on. The combination of single-cell analysis and microfluidics has the possibility to realize in situ, real-time, in vitro, and highly selective cell treatment and measurement. Thus, microfluidics for single-cell analysis has important academic significance and great application potential with no doubt. Although researchers have made many efforts in this field, lots of problems still need to be investigated.

With the rapid development of polymer-based microfluidic in recent years, it has already been proved to be powerful tools for single-cell analysis. As mentioned above, microfluidic has the advantages of highly analysis efficiency, easy operation, small sample and reagent consumption, and dimension matching between channel sizes and cell diameter. At the same time, polymer materials also show great superiority based on their variety, processability, and low cost.

Microfluidics fabricated with an appropriate polymer material is a sure way of multiplying the effectiveness of different manipulation, treatment, and analysis of specific cells. However, there is also an urgent need for improvement in polymer-based microfluidics for single-cell analysis. As an emerging technique, polymer-based microfluidics for single-cell analysis has not been widely adopted by the community of cell-biology researchers. Although engineering researchers have developed many methods for the manufacture of polymer-based microfluidics, most of these methods are still at the prototype stage. That is, users must undergo significant training before operation, and the microfluidics produced by these manufacturing methods are not completely stable. Basically, we can say that only the microfluidics fabricated with PDMS have a large number of application in cell analysis, which benefit from the widespread, robust, and simplified operation of the soft lithography method. Arguably, all round collaboration between cell biology and engineering researchers is necessary. On the one hand, the cell biology community can provide ideas and requirements for the integration of more traditional detection techniques and microfluidic devices. On the other hand, engineering researchers can design manufacturing methods that depend on specific requirements and pay more attention to the transfer of their methods to a robust microfluidic device.

Furthermore, the disconnect between the development and commercialization of microfluidics is also an inherent problem. Hence, to fill the gap between them is another primary mission for the future of polymer-based microfluidics. Polymer materials, especially commercial thermoplastics like PMMA, PS, etc., are especially suitable to use as a base material for commercial polymer-based microfluidics. Likewise, manufacturing methods that can be applied for the thermoplastics mentioned above, such as injection molding, hot embossing, laser ablation, etc., should be modified and optimized systematically for large-scale production. Microfluidics have the advantage over many techniques for early diagnostic methods, while polymer-based microfluidics have the potential to make self-service diagnostics at home affordable.

In a word, polymer-based microfluidics for single-cell analysis is a very important research field with great academic significance. With the mutual improvement and continuous progress of single-cell analysis and the manufacturing of polymer-based microfluidics, it will become more accurate, adoptable, and affordable in the near future. We believe that polymer-based microfluidics for single-cell analysis will promote the development of life sciences, benefiting all it touches finally.
